# Genetic diversity of *Plasmodium falciparum* reticulocyte binding protein homologue-5, which is a potential malaria vaccine candidate: baseline data from areas of varying malaria endemicity in Mainland Tanzania

**DOI:** 10.1186/s12936-025-05269-x

**Published:** 2025-01-27

**Authors:** Angelina J. Kisambale, Dativa Pereus, Salehe S. Mandai, Beatus M. Lyimo, Catherine Bakari, Gervas A. Chacha, Ruth B. Mbwambo, Ramadhan Moshi, Daniel A. Petro, Daniel P. Challe, Misago D. Seth, Rashid A. Madebe, Rule Budodo, Sijenunu Aaron, Daniel Mbwambo, Abdallah Lusasi, Stella Kajange, Samwel Lazaro, Ntuli Kapologwe, Celine I. Mandara, Deus S. Ishengoma

**Affiliations:** 1 National Institute for Medical Research, Dar Es Salaam, Tanzania; 2 Muhimbili University of Health and Allied Sciences, Dar Es Salaam, Tanzania; 3 Nelson Mandela African Institution of Science and Technology, Arusha, Tanzania; 4 University of Dar Es Salaam, Dar Es Salaam, Tanzania; 5 National Institute for Medical Research, Tanga Research Centre, Tanga, Tanzania; 6 National Malaria Control Programme, Dodoma, Tanzania; 7 President’s Office, Regional Administration and Local Government, Dodoma, Tanzania; 8 Directorate of Preventive Services, Ministry of Health, Dodoma, Tanzania; 9 Department of Biochemistry, Kampala International University in Tanzania, Dar Es Salaam, Tanzania

**Keywords:** Malaria, Malaria vaccine, *Pfrh5*, *Plasmodium falciparum*, Genetic diversity of *Pfrh5* gene, Mainland Tanzania

## Abstract

**Background:**

The limited efficacy of the two recently approved malaria vaccines, RTS,S/AS01 and R21/Matrix- M™, highlights the need for alternative vaccine candidate genes. *Plasmodium falciparum* Reticulocyte Binding Protein Homologue 5 (*Pfrh5)* is a promising malaria vaccine candidate, given its limited polymorphism, its essential role in parasite survival, a lack of immune selection pressure and higher efficacy against multiple parasites strains. This study evaluated the genetic diversity of *Pfrh5* gene among parasites from regions with varying malaria transmission intensities in Mainland Tanzania, to generate baseline data for this potential malaria vaccine candidate.

**Methods:**

This study utilized secondary data of 697 whole-genome sequences which were generated by the MalariaGEN Community Network. The samples which were sequenced to generated the data were collected between 2010 and 2015 from five districts within five regions of Mainland Tanzania, with varying endemicities (Morogoro-urban district in Morogoro region, Muheza in Tanga, Kigoma-Ujiji in Kigoma, Muleba in Kagera, and Nachingwea district in Lindi region). Wright's fixation index (F_ST_), Wright’s inbreeding coefficient (Fws), Principal component analysis (PCA), nucleotide diversity (π), haplotype network, haplotype diversity (Hd), Tajima's D, and Linkage disequilibrium (LD) were used to assess the diversity of the gene.

**Results:**

Of the sequences used in this study, 84.5% (n = 589/697) passed quality control and 313 (53.1%) were monoclonal (contained infections from a single strain of *P. falciparum*) and were used for haplotype diversity and haplotype network analysis. High within-host diversity (Fws < 0.95) was reported in Kigoma-Ujiji (60.7%), Morogoro-urban (53.1%), and Nachingwea (50.8%), while Muleba (53.9%) and Muheza (61.6%) had low within-host diversity (Fws ≥ 0.95). PCA did not show any population structure and the mean F_ST_ value was 0.015. Low nucleotide diversity values were observed across the study sites (mean π = 0.00056). A total of 27 haplotypes were observed among the 313 monoclonal samples and under-fives exhibited higher haplotype counts. The *Pf*3D7 was detected as Hap_1, which occurred in 16/313 (5.1%) monoclonal sequences. Negative Tajima's D values were observed among the parasite populations in all the study sites.

**Conclusion:**

Low levels of polymorphism in the *pfrh5* gene were observed based on low nucleotide and haplotype diversity, a lack of population structure and negative Tajima’s D values. This study provides essential data on the diversity of the *Pfrh5* gene indicating that it can be considered in the development of the next generation malaria vaccines. Robust and intensive studies of this and other candidate genes are crucial to support the prioritization of the *Pfrh5* gene for potential inclusion in a broadly cross-protective malaria vaccine.

## Background

Despite substantial efforts and resources which have been invested to support implementation of different interventions to control and eliminate malaria, the disease continues to pose a significant global health challenge [1]. The World Health Organization (WHO) reported about 249 million malaria cases and 608,000 deaths in 85 malaria-endemic countries in 2022, with most of the cases (93.6%) and deaths (95.4%) from the WHO African region [2]; and over 96.0% of the cases were due to *Plasmodium falciparum*. According to the 2023 World Malaria Report, Tanzania was among the 11 countries with the highest number of malaria deaths and cases in sub-Saharan Africa (SSA); it accounted for 3.2% of the global cases and 4.4% of global malaria deaths [2]. Over 93.0% of the population in Tanzania lives in areas where transmission occurs, and the entire population of Mainland Tanzania is considered at risk of malaria. However, the transmission rates and burden vary among and within regions, with high transmission in western, northwestern and southern regions [3].

Despite the efforts deployed in the past 2.5 decades, malaria control and elimination is still challenged by key biological threats including resistance to insecticides by mosquitoes, and anti-malarials by the parasites. Other threats include histidine rich protein 2/3 (*hrp2/3*) genes deletions which compromise the effectiveness of HRP2-based rapid diagnostic tests and the spread of invasive vector species, *Anopheles stephensi* [4–8]*.* There are also non-biological challenges and threat such as climate change, health system limitations and insufficient funding which hinder malaria control efforts as they diminish the effectiveness of or coverage and access to interventions [9, 10]. Additionally, the complex life cycle of the plasmodium parasite with developments in mosquito vector and human hosts pose significant challenges, particularly to interventions such as malaria vaccines [11, 12]. Thus, innovative tools are urgently needed and integrating vaccines into existing malaria control strategies is crucial for achieving malaria elimination. The success of vaccines in controlling, eradicating and eliminating other serious diseases like COVID-19 and smallpox underscores the potential of vaccines to enhance malaria control, elimination and eventual eradication [13, 14].

In the efforts to effectively control/eliminate malaria, the WHO approved two malaria vaccines, RTS,S/AS-01 in 2021 and R21/Matrix-M™ in 2023, for use in children from countries or areas with moderate to high malaria burden [15]. Both vaccines are subunit vaccines, targeting the *P. falciparum* circumsporozoite protein (*Pf*CSP). This protein is expressed in pre-erythrocytic stages on the surface of sporozoites and it is responsible for hepatocyte invasion [15]. Studies have demonstrated that the RTS,S/AS-01 vaccine exhibit greater efficacy against parasites with alleles matching the vaccine strains compared to diverse alleles in the targeted populations [16, 17]. Comparative diversity studies of the 3D7 *Pfcsp* gene revealed that only 0.2% to 5.0% of the vaccine strains align with the global *Pfcsp* gene [16]. Studies from different malaria-endemic countries including Tanzania [18], Ghana [19] China [20] and Myanmar [21], have demonstrated a significant level of genetic diversity in the *Pfcsp* gene, hence standing as one of the challenges for the efficacy of this vaccine. Contrary, studies have shown that the R21/Matrix-M™ vaccine had high efficacy (up to 75%) and safety in phase III trials, representing a significant advancement in the fight against malaria. However, the vaccine efficacy was not the same in all age groups as it was higher among younger children compared to older children [22]. These findings underscore the need for new malaria vaccines that can maintain high efficacy across diverse strains, and can be used in areas of varying endemicity and in all age groups.

**Fig. 1:**
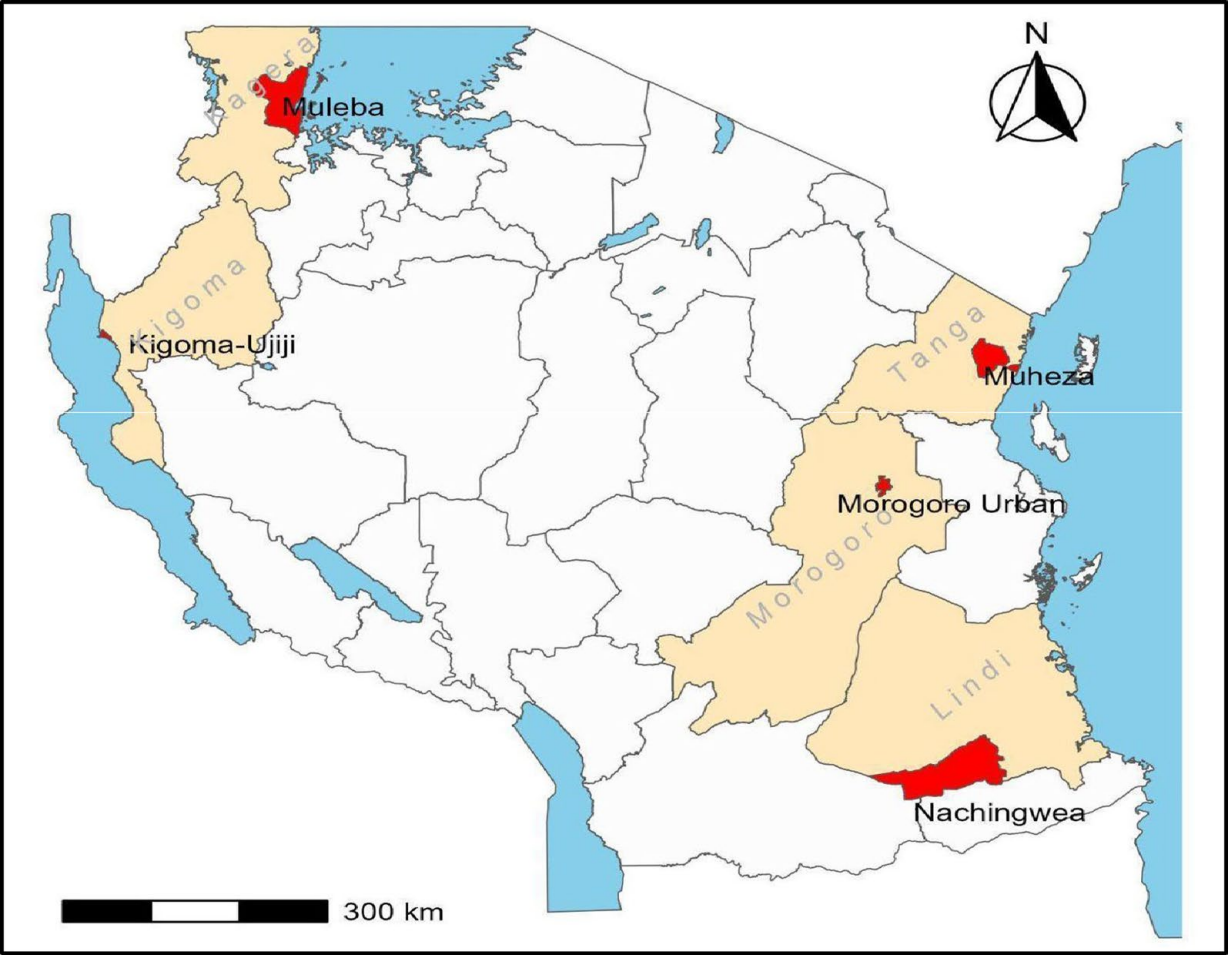
Map of Tanzania showing the five districts where sampling was undertaken (red colour)

The *P. falciparum* reticulocyte-binding protein homologue 5 (*Pfrh5*) complex stands out as a crucial target for developing an effective vaccine targeting the blood stage [23, 24]. *Pfrh5*, a 63 kDa protein is encoded by the gene PF3D7_0424100 (located on chromosome 4), and plays a key role in invasion of erythrocytes by the merozoites. The *pfrh5* forms a pentameric complex containing *P. falciparum* thrombospondin-related apical merozoite protein, *P. falciparum* cysteine-rich protective antigen and PfRH5-interacting protein post-release from the rhoptries [25]. This process facilitates *Pfrh5* expression on the merozoite surface during invasion, where its interaction with the receptor basigin (BSG) also known as CD147 via hydrogen bonds is vital for erythrocyte invasion [26]. Antibodies targeting the PfRH5-BSG invasion pathway have demonstrated a significant inhibition in the invasion of erythrocytes [27]. Studies have also highlighted the effectiveness of *Pfrh5* antibodies in inhibiting the growth of various *P. falciparum* strains, surpassing other antigens on vaccine development platforms [28, 29]. Moreover, *Pfrh5* exhibits relatively low genetic variation, with a limited number of non-synonymous mutations which have been identified in this gene [30, 31]. This conservation with minimal polymorphism sets *Pfrh5* apart from other prominent subunit vaccine candidates like apical membrane antigen-1 (AMA1) [32] and merozoite surface protein-1 (MSP1) [33].

Polymorphisms in genes related to malaria vaccine candidates are one of the key factors hindering the development of other malaria vaccines and need to be considered when selecting and optimizing any potential malaria vaccine candidate [34, 35]. Therefore, there is an urgent need to utilize the recently generated global multi-omics data of malaria parasites to identify highly conserved vaccine candidates, which will facilitate and expedite the development of highly efficacious vaccines that will be critical in the ongoing malaria elimination and eradication efforts. Before proceeding with vaccine development based on *Pfrh5* antigen, a thorough investigation and characterization of *Pfrh5* genetic diversity in regions with varying malaria endemicity and heterogeneous transmission patterns, such as Tanzania (which is an endemic country located in East Africa), are imperative [36]. Therefore, this study was conducted to evaluate the genetic diversity within the *Pfrh5* gene isolated from parasites collected in areas exhibiting varying levels of transmission intensities in Mainland Tanzania to generate baseline data of the level of polymorphism in this potential malaria vaccine candidate.

## Methods

### Study design and sampling

The study utilized data which were retrieved from an open dataset of the *P. falciparum* genome variation of the Genomic epidemiology of malaria network (MalariaGEN Pf7) [37]. The collection of samples which were used by MalariaGEN was conducted as part of the Pathogen Diversity Network Africa’s baseline surveys across 15 countries in SSA [38]. Sample collection was done in five districts located in five regions from 2010 to 2015 through different studies including, the therapeutic efficacy studies (TES) of children aged 6 months to 10 years, cross section surveys (CSS) of patients aged 6 months and above, and longitudinal and cross-sectional birth cohort studies (Fig. 1). The TES were conducted at Mkuzi health centre in Muheza district (Tanga region), Muheza designated district hospital (DDH) also in Muheza district (Tanga region), and Ujiji health centre in Kigoma-Ujiji district (Kigoma region) as previously described [39–41]. The CSS were conducted at Rubya DDH in Muleba district (Kagera region), Mkuzi health centre in Muheza district (Tanga region) and Nachingwea district hospital in Nachingwea district (Lindi region) [42]. The longitudinal and cross-sectional birth cohort studies were conducted at Muheza DDH (Tanga region) and Morogoro regional hospital in Morogoro-urban district (Morogoro region) through the mother and offspring malaria study as described earlier[43, 44]. Based on the 2020 stratification of malaria burden; Muheza and Morogoro-urban were classified as areas with moderate transmission of malaria while Nachingwea, Muleba and Kigoma-Ujiji were areas with high transmission intensity [45]. Similar data have also been used for studies of *csp* polymorphism and evidence of selection within these gene in Mainland Tanzania [18].

The current analysis involved a total of 697 samples that were collected as whole blood from the five districts in the five regions in Mainland Tanzania (Fig. 1). The number of samples from each site included 324 samples from Muheza district, 34 from Morogoro -urban, 199 from Kigoma-Ujiji, 79 from Nachingwea and 61 samples in Muleba district (Table 1). Parasite genomic DNA was extracted from leucocyte depleted samples using QIAamp DNA blood mini kits according to manufacturers’ instructions (Qiagen GmbH, Hilden, Germany). Genomic data were generated by whole genome sequencing (WGS) using illumina short reads which were done through the *P. falciparum* Community Project of MalariaGEN at the Wellcome Sanger Institute, UK as described earlier [35].

**Table 1:** Details of sequenced samples which were collected from the five districts of Mainland Tanzania and used for this study

Study site	Retrieved sequences	Passed filtering score	Monoclonal (%)	Polyclonal (%)
Morogoro urban*	34	32	15(46.9)	17(53.1)
Muheza**	324	297	182(61.3)	115(38.7)
Muleba**	61	52	27(51.9)	25(48.1)
Nachingwea**	79	65	32(49.2)	33(50.8)
Kigoma-Ujiji***	199	143	57(39.9)	86(60.1)
Total	697	589	313(53.1)	276(46.9)

^*^Sample collection in Morogoro-urban was done through the Mother and offspring Malaria study which was also conducted at Muheza DDH [43, 44]

^**^Samples from Muheza were collected through two therapeutic efficacy studies and a cross-sectional study which also covered Nachingwea and Muleba [39, 40, 42]

^***^Sample collection in Kigoma-Ujiji was done in a therapeutic efficacy study as previously reported [40, 41]

### Data retrieval and sequence acquisition

Metadata were available for the samples collected in all sites except Morogoro because of difficulties reaching the team which conducted the studies. WGS of 697 *P. falciparum* and sample information from each study site were mined from the database of the MalariaGEN *Plasmodium falciparum* Community (Pf7) project in variant call format (VCF). The *Pfrh5* genomic location, genomic length and the Pf3D7 reference sequence were obtained from the PlasmoDB database: https://plasmodb.org/plasmo/app/record/gene/PF3D7_0424100. The gene was extracted from chromosome 4 at position 1082005–1084464 using bcftools [46, 47].

Before progressing with downstream data analysis, the sequences were filtered to include only biallelic variants with a variant quality score log odds (VQSLOD) of ≥ 1. The Pf3D7 reference genome (Pf3D7_0424100) from PlasmoDB was indexed using Samtools v 1.18, and then Picard v 3.1.0 tool was used to create a sequence dictionary for the reference genome (https://broadinstitute.github.io/picard/). Sequences in the VCF file were aligned to the Pf3D7 reference sequence using MAFFT [48] and variants were called using vcftools [49].

**Fig. 2:**
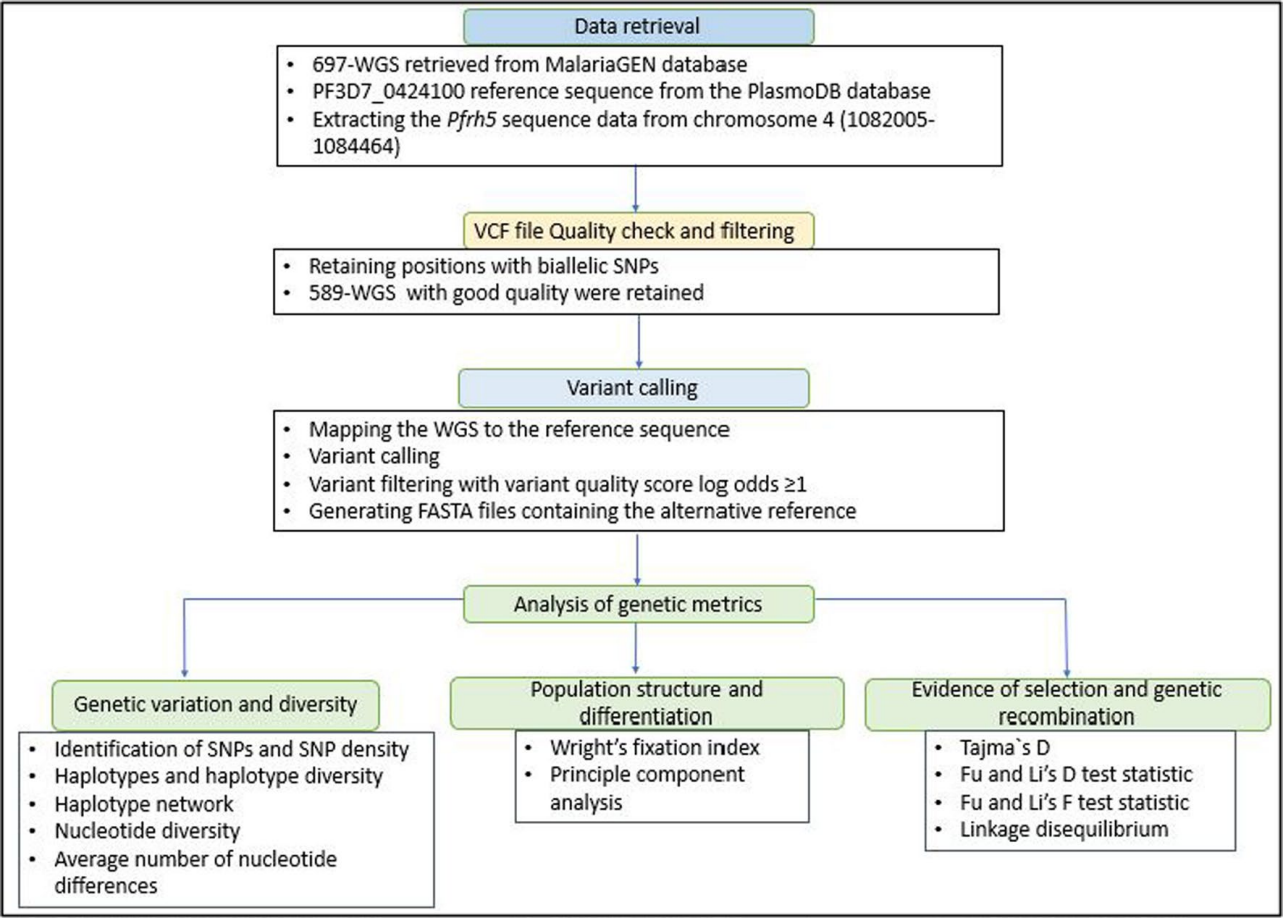
Summary of the process undertaken for data retrieval, processing and downstream analysis of the genetic metrics in the *Pfrh5* gene

Subsequently, the Genome Analysis Toolkit (GATK) 4.4.0.0 was used to index the processed VCF files and generate individual FASTA files with the alternative reference corresponding to the processed VCF files [50]. The individual FASTA files were then merged into one major file for subsequent downstream analysis. The biallelic variants that passed quality filter scores were used for further analysis [51]. Genetic metrics were subsequently analysed using DnaSP v 6.12 software and R v.4.4.0. The process of WGS data retrieval, extraction of *Pfrh5* gene, processing and analysis of genomic data was done as summarized in Fig. 2.

### Analysis of genetic metrics

#### Within host-parasite diversity

Within-host diversity was evaluated by estimating Wright’s inbreeding coefficient (Fws) for each population using the “moimix” package in R v 4.4.0 [52]. The Fws metric measured the degree of polyclonal infections, assessing the within-host diversity of *P. falciparum* in relation to local population diversity [53]. Fws ranges from 0 to 1, whereby a low Fws value indicates high degree of genetic diversity within the specific parasite populations compared to the overall population. Samples with a high Fws value (≥ 0.95) were classified as monoclonal (single strain) infections, while those with a low Fws value (< 0.95) were regarded as polyclonal infections (indicating that they were mixed strain infections) [54, 55]. A Pearson chi-square test was conducted in R v.4.4.0 to assess and determine the differences in Fws estimates among the different study populations, with a standard threshold of p < 0.05 indicating statistical significance.

### Genetic diversity within parasite populations

FASTA DNA sequences were generated from the processed VCF files using the GATK v 4.4.0.0, and were used to determine different genetic metrics that were used for assessing the genetic diversity of the *Pfrh5* gene within each parasite population using DnaSP v.6.12 software. These genetic metrics included the number of segregating sites (S), referred to as the position within the gene sequence where there are variations between alleles observed in a population, the number of haplotypes (H: A set of DNA variants along a chromosome that tend to be inherited together), and haplotype diversity (Hd); a measure of the variety of haplotypes present within a population. The number of haplotypes were also computed and compared among patients of different age groups in the four study sites of Kigoma-Ujiji, Muheza, Muleba and Nachingwea which had demographic data (such data were not available for Morogoro-urban district). The age groups were categorized as under-fives, school children aged 5-< 15 years and adults aged ≥ 15 years. Adegenet version 2.1.10 and ape version 5.7.1 packages in R were utilized to calculate and visualize SNP density using the fasta2bin and seg.sites functions [53, 56]. The determined SNP density was then plotted against the nucleotide positions along the gene with a window size range of 500 base pairs. Other genetic metrics included, singleton variable sites (Sn); for mutations appearing only once among the sequences, and Parsimony informative sites; (sites containing at least two types of nucleotides and at least two of them occurring with a minimum frequency which is required for evolutionary changes of a genome). Nucleotide diversity was used to measure the average number of nucleotide differences per site between any two randomly chosen DNA sequences within a population estimated as π. Violin plots were used to visually assess the median differences of nucleotide diversity between and among the populations, implemented in ggplot2 packages in R workflow. To examine the genetic connectivity among *Pfrh5* haplotypes across the five districts, the haplotype networking of 313 *Pfrh5* monoclonal sequences (53.1%) was analysed using NETWORK version 10.2, employing the Median-joining algorithm [57]. The sequence of Pf3D7_0424100, a reticulocyte binding protein homologue 5 (Pf3D7), was downloaded from the PlasmoDB database https://plasmodb.org/plasmo/app/record/gene/PF3D7_0424100 and used to compare its variation and clustering with those of natural parasite populations.

### Population structure and differentiation

To assess gene flow between parasite populations, genetic differentiation was estimated using the Wright Fixation Index (F_ST_) [58] using the PopGenome package in R v 4.4.0 workflow. The F_ST_ measures population differentiation due to genetic structure, and values of < 0.05 indicate minimal population differentiation or gene flow between pairs of populations [59]. Violin plots were also generated to visualize the differences in F_ST_ values among the specific study populations using the ggplot2 packages in R. Principal component analysis (PCA); a linear technique used for data visualization was also used to assess the population structure and it was implemented in R version 4.4.0. The PCA is used to infer population structures by identifying genetic differences and clustering of individuals based on genetic variation [60].

### Evidence of selection and genetic recombination

The neutrality tests were performed to determine whether the *Pfrh5* gene is under balancing or purifying selection. This was done using Tajma`s D statistical test to analyse the departure of the gene from neutrality theory based on allele frequency distribution in the gene. The analysis was performed in sliding windows with a window length of 100 base pairs and a step size of 25, utilizing the total number of mutations and excluding sites with gaps in a DnaSP v. 6.12 Software [61, 62]. Tajima's D is anticipated to be close to zero under neutral evolution. The positive values of Tajima's D indicate signals of departures from neutrality, which may be caused by balancing selection or a reduction in population size. Conversely, negative Tajma`s D values are indicative of a purifying selection or population growth following a bottleneck event [63].

To validate the observed values of signature of selection, Fu and Li’s D and F test statistics were also assessed. The Fu and Li’s D test statistic was used to assess the differences between the observed number of singletons (mutations appearing only once among the sequences), and the total number of mutations while the Fu and Li’s F test statistic measured the differences between the number of singletons and the average number of nucleotide differences between pairs of sequences [64, 65]. Additionally, Linkage disequilibrium (LD) was computed considering all the polymorphic sites to assess the level of non-random association between alleles. Genetic association between polymorphic sites and the effect of intragenic recombination on sequence polymorphism were assessed on the polymorphic sites using Zns and the ZZ statistics, respectively [66, 67].

## Results

### Baseline information

A total of 697 WGS were retrieved from the MalariaGEN Pf7 database. Of the retrieved sequences, 84.5% (n = 589/697) passed quality filtering scores and were subsequently used for downstream analysis. Among the sequences which passed the filtering scores, 50.4% (n = 297/589) were from Muheza district while Kigoma-Ujiji had 24.3% (n = 143/589), and the rest were from the three remaining districts, with 11.0% (n = 65/589) from Nachingwea, 8.8% (n = 52/589) from Muleba and 5.4% (n = 32/589) from Morogoro-urban district. Of the successfully retrieved sequences, 53.1% (n = 313/589) had monoclonal infections and were used for analysis of haplotype diversity and haplotype networks (Table 1). Of the 589 retained sequences, 77.4% (n = 456) had age information from the metadata database of the studies which contributed samples to the MalariaGEN dataset. Of these 456 samples, 69.5% (n = 317) were from under-fives, 19.5% (n = 89) were from school children (aged 5- < 15 years) and 11.0% (n = 50) were from adults aged ≥ 15 years. The distribution of the samples based on clonality in the different age groups and in the five districts is shown in Table 2.

**Table 2:** Distribution of clonality in different age groups among the sequenced samples from the five districts of Mainland Tanzania

Study site	Age group (years)	Available from metadata	Monoclonal n (%)	Polyclonal n (%)
Muheza	< 5	124	79 (63.7)	45 (36.3)
	5- < 15	50	24 (48)	26 (52)
	≥ 15	24	16 (66.7)	8 (33.3)
Muleba	< 5	21	10 (47.6)	11 (52.4)
	5- < 15	15	6 (40)	9 (60)
	≥ 15	16	11 (68.8)	5 (31.2)
Kigoma-Ujiji*	< 5	99	41 (41.4)	58 (58.6)
	5- < 15	44	16 (33.4)	28 (66.6)
	≥ 15	-	–	–
Nachingwea	< 5	31	16 (51.6)	15 (48.4)
	5- < 15	21	9 (42.9)	12 (57.1)
	≥ 15	11	5 (45.5)	6 (54.5)
TOTAL		456	**233 (51.1)**	223 (48.9)

^*^All the data from Kigoma-Ujiji were from children aged 0-10 years who took part in therapeutic efficacy study and there was no data of adults aged ≥ 15 years in this site

### Within-host genetic diversity

**Fig. 3:**
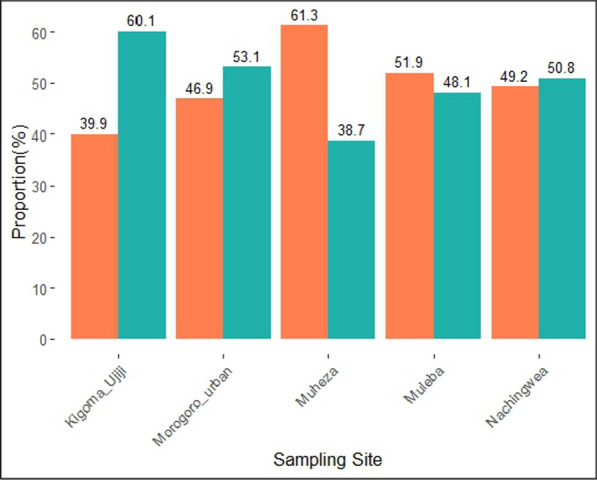
Bar plots showing the proportion of monoclonal (orange) and polyclonal (green) samples in the study districts

The degree of genetic diversity within each population was estimated through the wright's inbreeding coefficient (Fws), and showed that majority of the samples from Kigoma—Ujiji (60.1%, n = 86/143), Morogoro-urban (53.1%, n = 17/32) and Nachingwea (50.8%, n = 33/65) had polyclonal infections (Fws < 0.95). In contrast, most of the samples from Muleba (51.9%, n = 27/52) and Muheza (61.3%, n = 182/297) had monoclonal infections (Fws ≥ 0.95) (Table 1 and Fig. 3). The differences in the within-host diversity of the *Pfrh5* gene based on the percentages of polyclonal infections among the study sites were statistically significant (p < 0.001). In all study sites, the highest polyclonality was observed in school children (aged 5—< 15 years) while the largest proportion of monoclonal samples was observed in under-fives in Nachingwea and adults (aged ≥ 15 years) from Muheza and Muleba districts (Table 2).

### Genetic diversity among the parasite populations

**Fig. 4:**
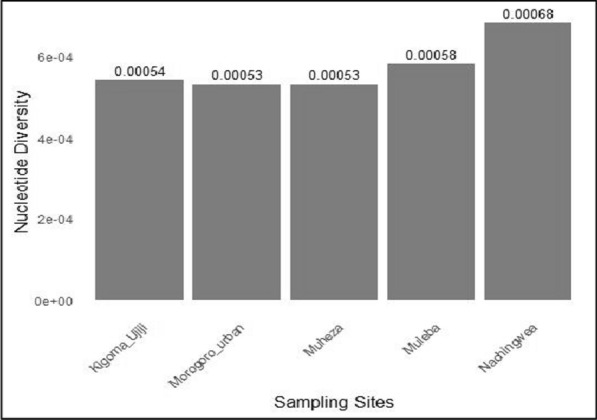
Bar plots showing nucleotide diversity in the *Pfrh5* gene among the parasite populations from the five districts

**Fig. 5:**
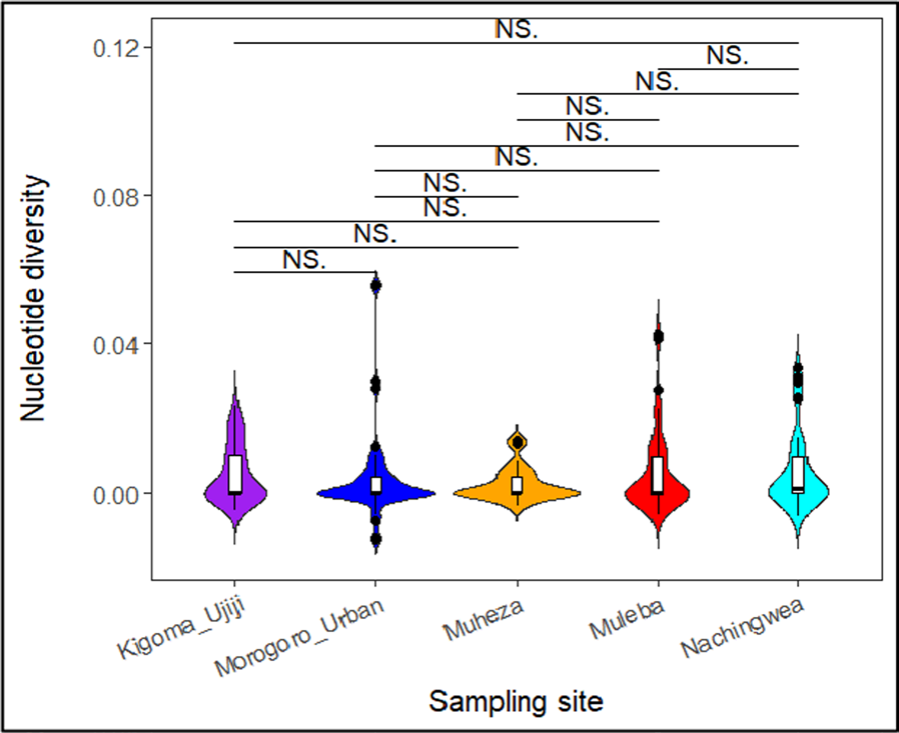
Violin plots showing the distribution of nucleotide diversity in the *Pfrh5* gene across the five populations represented with different colours. The black lines inside the box plot indicate the median nucleotide diversity. Black horizontal lines on top of the figures show the populations being compared, NS indicates that there was no-significant difference between or among the populations

**Fig. 6:**
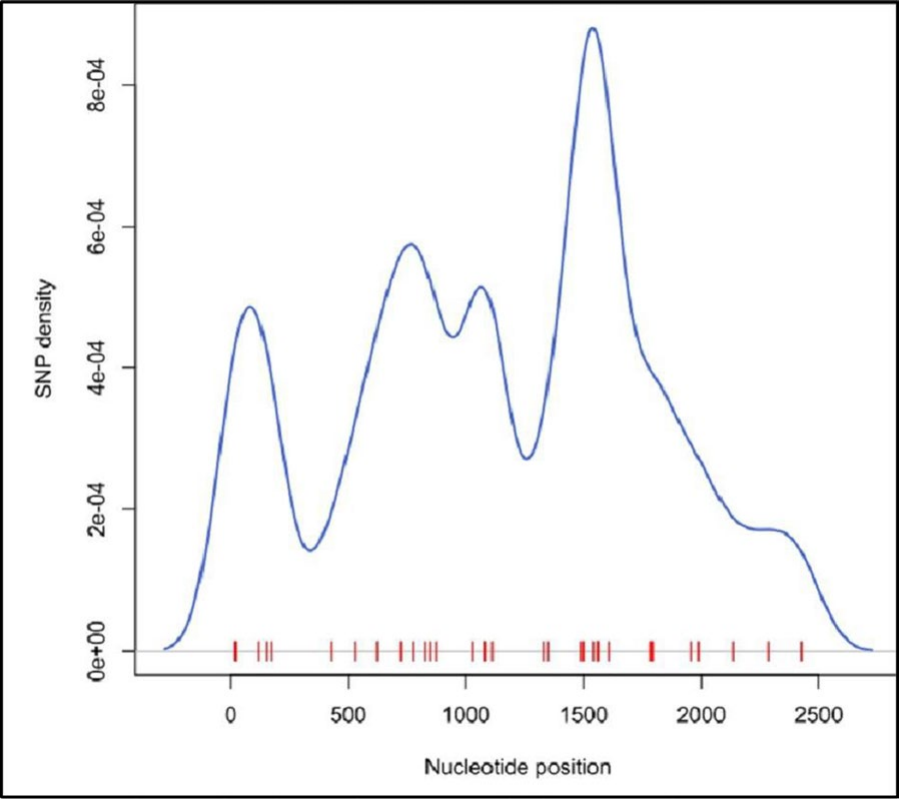
SNP density plot showing the positions of the SNPs along the *Pfrh5* gene. The blue line shows the SNP density, while the red bars depict the precise locations of the SNPs

The nucleotide diversity values were consistently low across all study sites ranging from 0.00053 in Muheza and Morogoro-urban to 0.00068 in Nachingwea (Fig. 4). When nucleotide diversity was visualized using violin plots, no significant differences were detected in the levels of nucleotide diversity among the populations (Fig. 5). Furthermore, the overall average pairwise number of nucleotide differences in the *Pfrh5* gene was 1.4, and it ranged from 1.3 in Muheza, Kigoma-Ujiji, and Morogoro-urban to 1.7 in Nachingwea (Table 3). A total of 40 SNPs were identified across the entire gene and the SNP density plot showed relatively higher occurrence of SNPs at the nucleotide positions around 1500 and 2000 base pairs (Fig. 6).

**Table 3:** Measure of *Pfrh5* DNA sequence polymorphisms among *P. falciparum* isolates in each study site

Polymorphisms	Study sites				
	Muheza	Morogoro urban	Nachingwea	Muleba	Kigoma-Ujiji
n	297	32	65	52	143
No. of segregating sites	16	7	20	10	15
Synonymous mutation	2	0	4	1	2
Non synonymous mutation	14	7	16	9	13
Sn	7	5	15	5	4
Parsimony informative sites	9	2	5	5	11
k	1.4	1.3	1.7	1.4	1.3
h	18	5	8	5	7
F_ST_	0.006	0.004	0.026	0.017	0.020
Hd	0.63	0.63	0.67	0.62	0.44
Tajma`s D	− 1.21	− 0.74	− 1.84	− 1.02	− 1.35
LD	0.06	0.32	0.16	0.12	0.22
Fu and Li's D	− 2.8	− 2.3	− 4.3	− 1.7	− 0.7
Fu and Li's F	− 2.5	− 1.9	− 3.9	− 1.6	− 1.1

n = number of sequences, S- number of segregating sites, Sn- number of singletons, k = Average number of nucleotide differences, F_ST_ = Wright Fixation Index, h = Number of haplotypes, Hd = Haplotype diversity, LD = Linkage disequilibrium

**Fig. 7:**
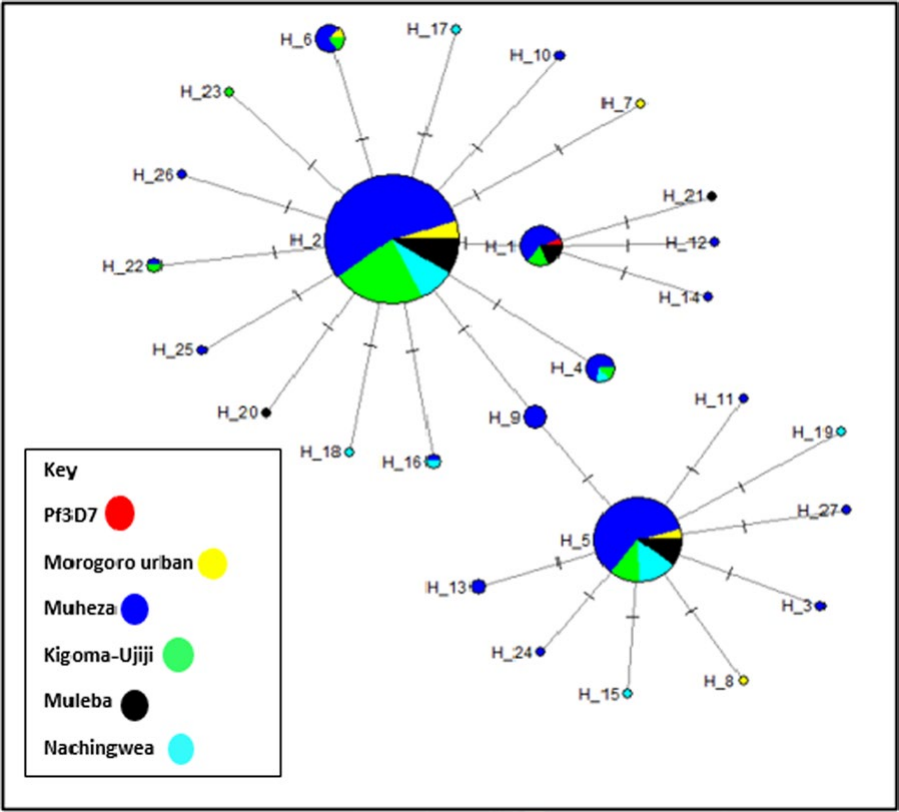
Median-Joining (MJ) haplotype network of the 27 haplotypes from *Pfrh5* sequences of *P. falciparum* in five Tanzanian regions. The size of the circle corresponds to the number of individual samples for each haplotype, while the colours represent the geographic distribution of each haplotype

**Fig. 8:**
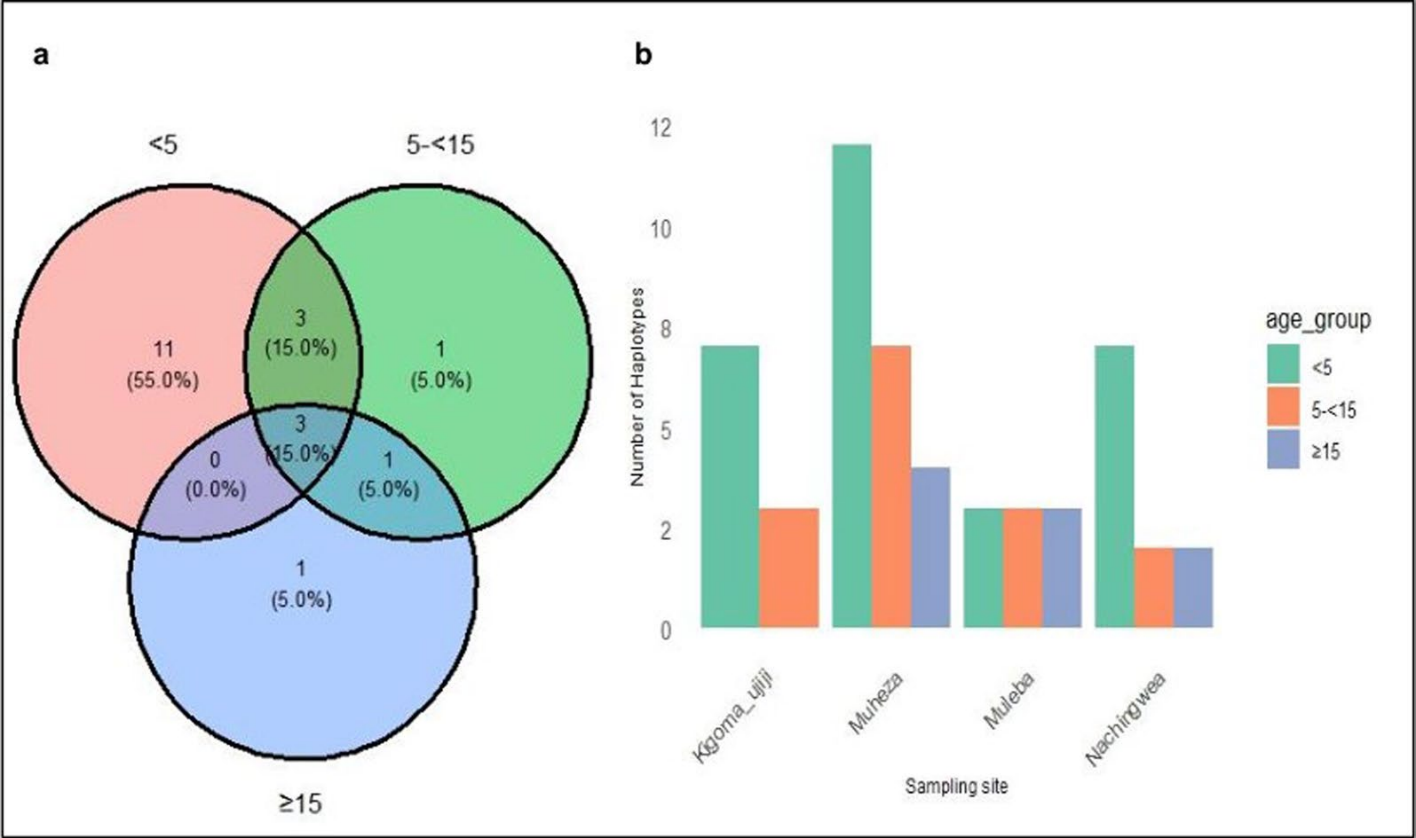
Haplotype distribution in the *Pfrh5* gene among patients of different age-groups, overall (**a**) and per district (**b**)

To assess the extent of genetic diversity and similarity within and between the five populations, the diversity in the *Pfrh5* gene was investigated and summarized in a median-join haplotype diversity network. In total, 27 haplotypes were observed among the 313 (53.1%) monoclonal *Pfrh5* sequences. Two haplotypes, Hap_2 (n = 185) and Hap_5 (n = 71) accounted for 81.8% (n = 256/313) of the sequences and were shared across all five regions, suggesting genetic closeness and conserved nature of the gene among these populations. Haplotype 2 was the most common *Pfrh5* haplotype, representing 59.1% (185/313) of the isolates. The sequence of Pf3D7 was identified in Hap_1, and this haplotype occurred in 16/313 (5.1%) samples, which were from Muheza (62.5%, n = 10/16), Kigoma-Ujiji ( 18.8%, n = 3/16), and Muleba (18.8%, n = 3/16) (Fig. 7**)**. Analysis of haplotypes in sample sequences with age-specific information revealed a total of 20 haplotypes, and the distribution of haplotypes among the age-groups is presented in Fig. 8a. Among these haplotypes, 13 (65.0%) were unique to specific age groups, while 7 (35.0%) were shared between two or across all age groups. Under-fives exhibited the highest number of haplotypes (55%, n = 11/20) compared to other age groups. Notably, 15%, (n = 3/20) of the haplotypes were shared across all the age groups. When analysed per district, under-fives continued to have a higher haplotypes count compared to other age groups across all the districts, except in Muleba where individuals from all age groups exhibited the same number of haplotypes (Fig. 8b). The haplotype diversity values (Hd) estimated among the monoclonal sequences were 0.63 in Muheza, 0.62 in Muleba, 0.63 in Morogoro urban, 0.67 in Nachingwea and 0.44, in Kigoma-Ujiji. However, the differences in haplotype diversity among the sites were not statistically significant (P = 0.406).

### Population structure, differentiation and evidence of selection

**Fig. 9:**
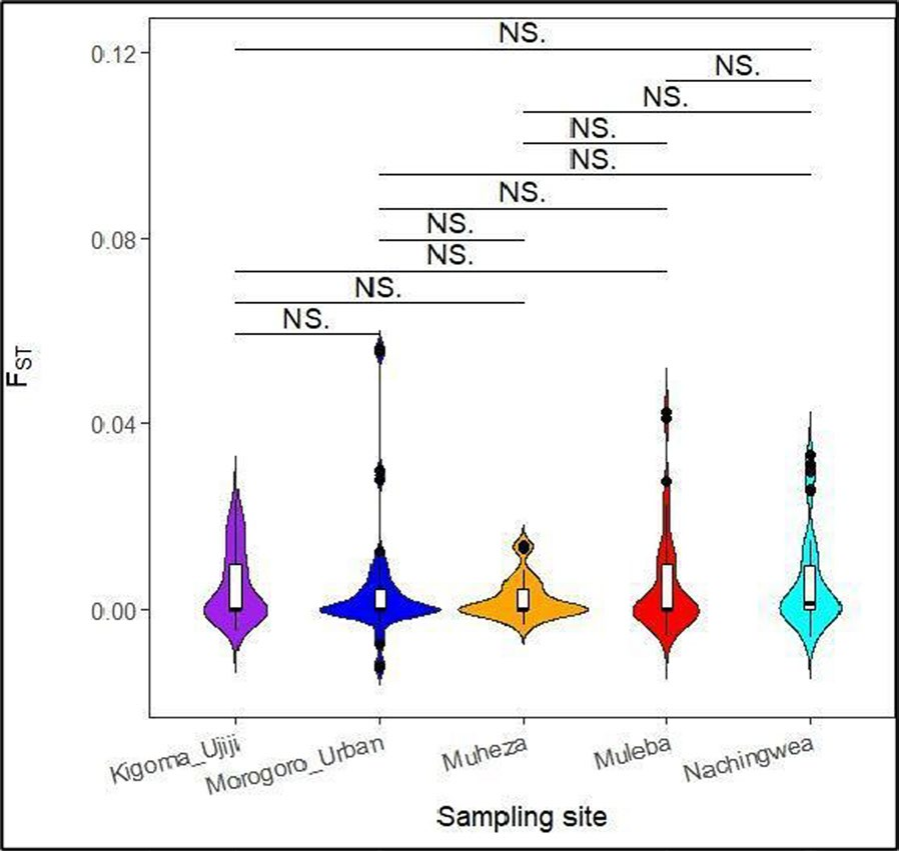
F_ST_ violin plots showing no significant difference F_ST_ values in the *Pfrh5* gene across the study populations. Black horizontal lines on top of the figures indicate the populations which were compared while he black lines inside the box plot indicate the median F_ST_ values, NS = no significant difference between or among the populations

**Fig. 10:**
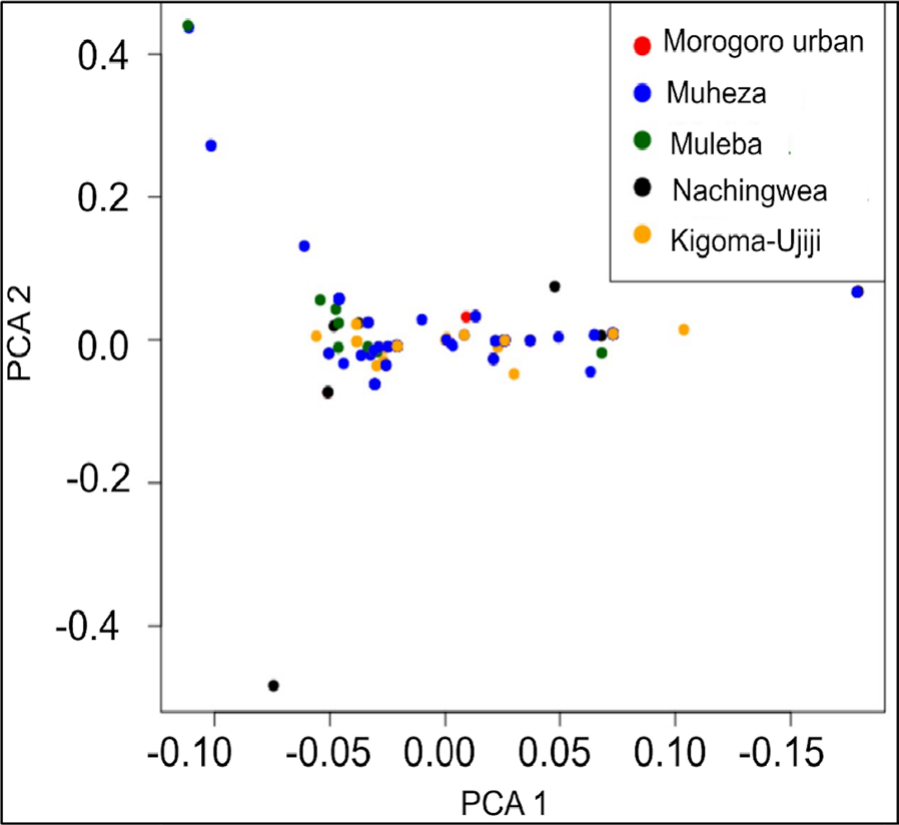
Plots of first (PC1) and second (PC2) Principal components of *Pfrh5* sequences in the five districts showing no population structure

Genetic differentiation which was estimated through the Wright Fixation Index (F_ST_) indicated that the mean F_ST_ among all sampled populations was 0.015. The F_ST_ values per site were 0.006, 0.017, 0.004, 0.026, and 0.020 in Muheza, Muleba, Morogoro-urban, Nachingwea and Kigoma-Ujiji, respectively (Table 3). These values indicate that there is low genetic differentiation among the populations. The violin plots showed that there were no significant differences in the genetic differentiation of the *Pfrh5* gene, as estimated by F_ST_ across all the five populations (Fig. 9). Principal component analysis did not show any population structure among parasite isolates in the sampled populations (Fig. 10).

The Tajma`s D values were negative across all sites indicating patterns consistent with purifying selection, with values of − 1.21 in Muheza, − 0.74 in Morogoro-urban, − 1.84 in Nachingwea, − 1.02 in Muleba and − 1.35 in Kigoma-Ujiji. However, the results were statistically significant only for Nachingwea (P < 0.05). The observed signatures of selection were further confirmed by the Fu and Li's D and Fu and Li's F test statistics where the values were negative in all the study sites (Table 3). The overall Linkage disequilibrium (LD) values for the *Pfrh5* gene assessing the non-random association of alleles in all the sequences was 0.11. The highest LD value (0.32) was in Morogoro-urban while the lowest LD value was observed in Muheza (LD = 0.06) (Table 3). The overall statistical values of the genetic association between polymorphic sites (Zns) and the effect of intragenic recombination on sequence polymorphism (ZZ) were 0.01 and 0.10, respectively.

## Discussion

Malaria endemic countries including Mainland Tanzania, face challenges in controlling and eliminating malaria due to the recent epidemiological transition. To achieve the global malaria elimination and eradication goals, it is crucial to consider the development of an effective vaccine alongside the existing interventions. However, high polymorphisms, low immunogenicity and limited efficacy of selected parasite proteins against diverse parasite strains make vaccines ineffective. The *Pfrh5* gene*,* a crucial component for *P. falciparum*'s invasion ligands that bind the receptors on human red blood cells, has been identified as an attractive vaccine candidate [68, 69]. However, limited data on the *Pfrh5* sequence polymorphisms necessitates population-specific studies on its sequence diversity to guide further development of an effective vaccine. This study evaluated the genetic diversity, population structure, and selection signatures within the *Pfrh5* gene from malaria parasite populations from five districts of Mainland Tanzania located in regions with varying endemicity. Overall, the findings indicate that the *Pfrh5* gene is relatively conserved and the negative population genetic tests suggest that the parasite population has limited capacity to accumulate and retain mutations.

Genetic diversity which was assessed based on estimates of within infection genetic diversity (Fws) showed that the majority of samples from Muheza and Muleba had low diversity with monoclonal infections as they had a high proportion of samples with Fws ≥ 0.95. Of the two districts, Muheza is located in an area of moderate transmission and high monoclonality in these districts reflects the high level of inbreeding among parasite populations within the study area. Higher within-host parasite diversity was observed in Morogoro-urban, Kigoma-Ujiji and Nachingwea where most of the samples had high level of polyclonality with Fws < 0.95. These districts (except Morogoro urban) are located in areas with high transmission [45] and this level of polyclonality is similar to what has been reported in areas with high transmission intensities of malaria [70]. Previous studies have reported a strong positive correlation between polyclonal infections and transmission intensity [70–72]. Such high levels of polyclonality and in areas of high transmission intensities are indicative of a high potential for recombination and outcrossing events in the population as previously reported [73, 74]. The high level of genetic diversity in high-transmission areas has been attributed to high proportions of infected individuals who usually carry polyclonal infections in contrast to low-transmission areas where infections are often monoclonal [75]. Thus, high levels of polyclonality among parasites in Kigoma-Ujiji and Nachingwea may be due to frequent infections with multiple clones, consistent with high malaria transmission intensities. The reasons for high levels of polyclonal infections in the urban district of Morogoro with moderate transmission are not clearly known but could be possibly due to importation of parasites from surrounding rural areas with high transmission. Previous studies have reported high levels of imported parasites in areas of low transmission due to human movements [76, 77]. However, the causes of high levels of monoclonal samples from Muleba which is located in an area with high transmission intensities are not clearly known and will need to be re-assed based on the data which are being generated by other studies (D. S. Ishengoma, pers. commun.).

The study observed very low nucleotide diversity across all study sites. This limited diversity likely results from the important function of the *Pfrh5* gene in the parasite's binding and invasion of erythrocytes. Given that *Pfrh5* is essential for invasion, significant structural changes due to mutations could impair the parasite's ability to infect red blood cells [36]. Consequently, the parasite avoids or restricts mutations in this gene, maintaining its ability to invade erythrocytes and this leads to low genetic diversity. Studies also suggest that very low values of nucleotide diversity are usually indicative of a very recent common ancestor to all sequences, as this might be expected from a hard sweep or a recent, strong bottleneck. A study of evolutionary events on human malaria suggested that a short region on chromosome 4, which encodes two essential invasion genes including the *Pfrh5* was horizontally transferred into a recent *P. falciparum* ancestor, an event that is similar to a very recent bottleneck [78]. The mean nucleotide diversity of this gene was extremely low (0.00056) compared to the nucleotide diversity seen in a well-known vaccine candidate gene, *Pfcsp* (0.0027) conducted in the same geographical areas [18]. Similar studies conducted in Mali and India also reported low average nucleotide diversity values in the *Pfrh5* gene (**π** = 0.00061 and 0.0007 in Mali and India respectively) [79, 80].

The identification of SNPs revealed low levels of polymorphism in the gene although the genomic regions around 1500 and 2000 base pairs exhibited relatively higher SNP counts. High SNP counts within these genomic regions suggest that they are potential hotspots for genetic variations within the gene. This may result from random mutations occurring within the genetic structure of *P. falciparum *[58]. Understanding the specific role of these genomic regions is essential for the efforts to develop a malaria vaccine based on the *Pfrh5* gene. Further investigations, such as structural studies of this gene, are necessary to clarify the implications of these findings and their relevance to vaccine design. Although the genomic regions around 1500 and 2000 base pairs exhibited a relatively higher SNP count, the overall gene displayed low levels of polymorphism, reflecting strong purifying selection. This selection preserves the gene's essential role in the parasite's survival while still allowing for occasional adaptive mutations. Low levels of polymorphisms were also reported in a study conducted in Mainland Tanzania, where they identified 12 polymorphic sites in the *pfrh5* gene, supporting the evidence that the *pfrh5* gene is conserved and makes it an attractive blood stage malaria vaccine candidate [23]. Conversely, these regions with high SNP density might reflect areas with high rate of mutation possibly due to their critical role in the parasite survival. However, continuous monitoring is warranted to assess whether the occurrence of these SNPs might affect vaccine efficacy.

This study also observed genetic similarity among the populations from the five sites, and a lack of population structure due to low F_ST_ values (F_ST_ < 0.05) and PCA. These findings suggest a high level of gene flow between the study populations and it is suggestive that interbreeding (which involves mating of parasites from different populations to produce offsprings) occurs more freely among the populations, implying the rapid spread of any introduced allele among *P. falciparum* populations. The low F_ST_ values and a lack of population structure could likely be attributed to human movements to different geographical areas. Migration of malaria infected humans increases the likelihood of gene flow leading to the spread of malaria related genetic traits in different populations regardless of their proximity. As a result, populations become more genetically similar to each other and the absence of barriers to gene flow leads to a homogenous population. Human movements between and among the study sites has been reported in other studies (Pereus et al., pers. commun.) and should have contributed to high gene flow among the parasite populations [81, 82]. Additionally, movements of parasites and connectedness should enable free parasite migration leading to complex parasite structure that share some of the identical or closely related genetic features on the background of the local genomic architecture. This complex mixing of parasites may explain the lack of population structure of the *pfrh5* gene observed in this study. Previous studies have highlighted the influence of human population mixing on promoting gene flow among *P. falciparum* isolates which likely increases the likelihood of self-fertilization and sporadic expansion of genetically identical parasites [83, 84]*.* Furthermore, the lack of population structure could also be related to the fact that the *Pfrh5* gene is highly conserved with limited polymorphisms within and among populations [31]. Also, the observed low values from other genetic metrics including nucleotide diversity, haplotype diversity and signatures of purifying selection suggest that the gene is highly conserved and these may have contributed to the low F_ST_ values and the absence of population structure among the studied populations. Similar findings of low genetic differentiation of the *Pfrh5* gene due to a lack of population structure were also reported in previous studies conducted in Kenya and Senegal [85, 86].

The haplotype network analysis of all sample with monoclonal sequences reported 27 different haplotypes with only one haplotype identical to the Pf3D7 reference strain. Out of the 27 haplotypes, two major haplotypes were distributed across all five districts while other haplotypes were shared among sites with possible indication of the circulation of alleles across different geographical settings. Although some other haplotypes occurred independently in each site, they did not contribute significantly to the genetic diversity within the gene as the gene remains conserved as indicated by low F_ST_ values and PCA results which showed limited differentiation among isolates from the sampled populations. The observed similarity of the haplotypes highlights the conserved nature of the *Pfrh5* gene, underscoring its crucial role in the parasite's survival, particularly in erythrocyte invasion. Consequently, the limited diversity in *Pfrh5* haplotypes is beneficial for developing *Pfrh5*-based vaccines, as it suggests that a single vaccine formulation could potentially offer broad protection against diverse strains of *P. falciparum* [87]. The observed relatedness of *Pfrh5* haplotypes indicates limited genetic variability of the gene across different parasite populations. Similar findings were also observed in a study conducted in Nigeria which compared the genetic diversity of the two vaccine candidate antigens (*pfrh5* and *P. falciparum* cell traversal ookinetes and sporozoites (*Pfceltos*)). The study reported that there were low variations of haplotypes in the *Pfrh5* gene as indicated by a shorter haplotype network and low haplotype diversity compared to the *Pfceltos* gene and suggested that the *Pfrh5* gene has the potential of being a more effective subunit vaccine because of its conserved nature [88]. Furthermore, the assessment of haplotype distribution by age groups revealed a remarkable finding because under-fives seemed to harbour more haplotypes compared to other age groups. The relatively higher number of haplotypes observed in under-fives could be a reflection of the levels of exposure to malaria and immune responses unique to this age group as young children are considered to be the vulnerable group due to their developing immune system and a lack of acquired immunity [89, 90]. Furthermore, the observation that 15% (n = 3/20) of the haplotypes were shared across all age groups indicates the circulation of alleles across different age groups, suggesting the presence of conserved regions within the *Pfrh5* gene. The age-dependent distribution of haplotypes with more haplotypes in under-fives, confirms the role of immunity in haplotype selection [91]. This, however, needs to be further investigated, considering the small sample size in each age group in some districts, as these observations could be influenced by a large proportion of under-fives who accounted for 69.5% of all the enrolled participants in these studies. However, these findings are comparable to those of a previous study from Uganda which assessed the age-distribution of haplotypes in the *Pfcsp* gene in which the majority of haplotypes were observed in under-fives [91].

Tajma`s D analysis revealed negative values in all the study sites suggesting a purifying selection on this gene. This was further confirmed by the Fu and Li’s D and F test statistics, where negative values were observed in all study sites using both tests, with significant results for both tests in Muheza (P < 0.05) and Nachingwea (P < 0.02). These negative population genetics summary statistics are indicative of an excess of rare variants in the *Pfrh5* gene suggesting that it is likely undergoing purifying selection and/or population expansion, both of which limit its capacity to accumulate and retain mutations. Stemming on the pivotal role of the *Pfrh5* gene on parasite`s ability to invade the host cells through erythrocyte invasion, any mutations that significantly alter the structure and function of the *Pfrh5* gene could affect the parasite`s ability to survive and reproduce thus these mutations are not favored, instead they are quickly eliminated in the population hence the domination of rare variants which are consistent with the observed negative Tajma`s D values. However, the Tajma`s D results were not statistically significant in the sites of Muheza, Morogoro -urban, Muleba and Kigoma-Ujiji suggesting weak evidence of purifying selection. In contrast, the results were significant in Nachingwea pointing to strong evidence supporting this interpretation. The observed results align with previous studies that reported rare variants in the *Pfrh5* gene and showed most sequences had a negative Tajima’s D value, suggesting a historical expansion of the parasite population [83, 85]. This observation is in contrast to other malaria vaccine candidates such as the MSP1, thrombospondin-related adhesion protein (TRAP) and CSP which have been reported to exhibit a balancing selection [18, 92].

Further, the assessment of non-random association of alleles at multiple sites through linkage disequilibrium revealed moderate levels of non-random association between alleles (LD = 0.1138). The limited LD is likely due to the genetic architecture of the *Pfrh5* gene which is dominated by the presence of rare variants that occur at high frequency possibly facilitated by the action of purifying selection [80]. Similar findings reporting an excess of rare variants in the *pfrh5* gene were observed in Kenya [85]. The essence of purifying selection together with the lack of population structure observed, suggests that the *Pfrh5* gene has the potential of being an effective vaccine candidate [93].

This study had some limitations which may limit the generalization of the findings. Firstly, the study was conducted in selected regions rather than covering the entire country. As a result, the findings may not fully represent the national context, potentially leading to biased conclusions. Secondly, the study used genetic data only and could not assess the gene using multi-omics data such as proteomic, metabolomic and other omics data. The study could not also perform immunological assessments of the gene in the selected areas, evaluate the role of PfRH5 proteins in the life cycle of *P. falciparum*, or examine its expression across different parasite strains; factors that are critical for vaccine development. In addition, the study used secondary data, which limited the availability and reliability of key metadata such as age distribution at certain sites. Although the study provides baseline findings of the gene, future studies are recommended to explore the PfRH5's role, immunogenicity, and strain-specific expression for vaccine development, and also incorporating primary data collection and encompass broader geographical areas to ensure more comprehensive and representative results.

## Conclusion

This study assessed the genetic diversity of the *Pfrh5* gene in areas with varying levels of malaria endemicity and found the gene to be conserved, as it exhibited low nucleotide and haplotype diversity, lacked population structure and had negative Tajma`s D values as evidence of purifying selection. These findings suggest that there is high gene flow and genetic exchange within the *Pfrh5* gene. Also, the *Pfrh5* gene is under selective pressure due to its crucial role in parasite survival and may exhibit limited genetic variation across populations. This study provides important evidence on the low genetic diversity of the *Pfrh5* gene and support for the gene to be considered in the design of next generation malaria vaccines. In the future, more studies will be needed to establish if *Pfrh5* could be included in a multi-antigen vaccine targeting sporozoites, merozoites, and transmission stages, or if it could be administered alongside the RTS,S or R21/Matrix-M^TM^ vaccines. Moreover, comprehensive and intensive studies across additional sites and incorporating other genetic metrics that were not assessed in this study are essential to further support the prioritization of this gene for potential inclusion in a broadly cross-protective malaria vaccine.

## Data Availability

No datasets were generated or analysed during the current study.

## Abbreviations

AMA1: Apical membrane antigen -1
CSP: Circumsporozoite protein
CSS: Cross sectional survey
DDH: District designated hospital
LD: Linkage disequilibrium
MSP 1: Merozoite surface protein 1
NIMR: National institute for medica research
PCA: Principal component analysis
PfRH5: Plasmodium falciparum reticulocyte binding homologue 5
SSA: Sub-Saharan Africa
TES: Therapeutic efficacy studies
TRAP: Thrombospondin-related adhesion protein
VCF: Variant call format
WGS: Whole genome sequence
WHO: World Health Organization
